# Comparison of heart failure risk and medical costs between patients with type 2 diabetes mellitus treated with dapagliflozin and dipeptidyl peptidase-4 inhibitors: a nationwide population-based cohort study

**DOI:** 10.1186/s12933-020-01060-1

**Published:** 2020-06-22

**Authors:** Jong-Mi Seong, Jong Joo Kim, Hae Jin Kim, Hyun Soon Sohn

**Affiliations:** 1 Research Institute for Pharmaceutical Sciences, Ewha Womans University, Seoul, 03760 Republic of Korea; 2 Pharmaceutical Information Research Institute, CHA University, Seongnam, 13488 Republic of Korea; 3 Department of Endocrinology and Metabolism, Ajou University School of Medicine, 206, World cup-ro, Yeongtong-gu, Suwon, 16499 Republic of Korea; 4 College of Pharmacy, CHA University, 335 Pangyo-ro, Bundang-gu, Seongnam-si, Gyeonggi-do 13488 Republic of Korea

**Keywords:** Dapagliflozin, Dipeptidyl peptidase-4 inhibitors, Heart failure, Type 2 diabetes mellitus, Direct medical costs

## Abstract

**Background:**

Dapagliflozin is one of the novel glucose-lowering agents, which has recently been reported to reduce the risk of hospitalization for heart failure (hHF). The present study aimed to compare the differences between the risk of hHF after using dapagliflozin and dipeptidyl peptidase-4 inhibitors (DPP-4i) as second-line drugs for the treatment of type 2 diabetes mellitus using the latest nationwide population data in Korea. Additionally, we aimed to examine the impact of clinical outcomes on direct medical costs in the two groups.

**Methods:**

The present population-based, retrospective cohort study was conducted using the nationwide claims data between September 01, 2014 and June 30, 2018. New users of dapagliflozin and DPP-4i were identified from the database and the differences in patients’ characteristics between the two groups were analyzed using propensity score-weighted analysis. Cox proportional hazards regression analysis was used to estimate the risk of hHF. A simple model was used for the estimation of direct medical costs for 3 years.

**Results:**

In total, 23,147 patients in the dapagliflozin group and 237,187 patients in the DPP-4i group were selected for the analysis. The incidence rates of hHF were 3.86 and 6.79 per 1000 person-years in the dapagliflozin and DPP-4i groups, respectively. In the entire study population, the hazard ratio for hHF in the dapagliflozin group compared to the DPP-4i group was 0.58 (95% confidence interval 0.46–0.74), with 0.55 (95% confidence interval 0.41–0.74) among patients with underlying cardiovascular disease and 0.66 (95% confidence interval 0.46–0.95) among patients without underlying cardiovascular disease. The direct medical costs were $57,787 lower in the dapagliflozin group than in the DPP-4i group for 3 years.

**Conclusions:**

This study showed that dapagliflozin lowers the risk for hHF and subsequently reduces direct medical costs compared to DPP-4i. The protective effect against hHF was more evident among patients with underlying cardiovascular disease.

## Background

Type 2 diabetes mellitus (T2DM), a classic metabolic disorder, is consistently on the rise globally and is highly prevalent in Korea, with a prevalence of 14.4% among adults aged ≥ 30 years and 29.8% among adults aged ≥ 65 years as of 2016 [1]. Diabetes mellitus (DM) is known to exacerbate the clinical state of patients with heart failure (HF) and is associated with all-cause and cardiovascular mortality [2]. Further, HF with reduced ejection fraction (HFrEF) is used as an independent predictor of the treatment outcomes in T2DM [3, 4]. The percentage of patients with HF among the T2DM participants recruited in recent clinical trials ranged from 10 to 28% [5–12]. T2DM is known as an independent risk factor for HF incidence [13], and the incidence of HF was 2.5 times higher in the T2DM group than the non-T2DM group in a retrospective cohort study that followed-up with the patients for 72 months [14]. Therefore, HF management in patients with T2DM is a clinically important issue.

In general, current guidelines for T2DM treatment recommend metformin as the first-line therapy, and if appropriate blood glucose regulation is not achieved with monotherapy, a combination therapy with additional drugs is recommended [15–17]. Recently updated guidelines recommend sodium glucose co-transporter 2 inhibitors (SGLT-2i) or glucagon-like peptide 1 (GLP-1) receptor agonist treatment as first-line in patients with established cardiovascular disease (CVD) and newly diagnosed T2DM [18]. In Korea, more than 70% of the patients with DM were prescribed combination therapy consisting of more than two drugs in 2016, among which, 56% of the combinations involved metformin and dipeptidyl peptidase-4 inhibitors (DPP-4i) [1]. This suggests that DPP-4i is the second most common drug used after metformin. On the other hand, the use of SGLT-2i, a recently introduced DM medication beginning with dapagliflozin, is consistently on the rise since their inclusion in the health insurance coverage in Korea. Though only 3% of the patients prescribed two-drug therapy were prescribed with metformin and SGLT-2i in 2016 [1], the use of SGLT-2i, owing to the drug’s cost, in the following 3 years increased fourfold than that in 2016 [19].

However, a controversy regarding the potential association between DPP-4i, which is the most widely used drug as a second-line therapy, and HF exists. While alogliptin, vildagliptin, sitagliptin, and linagliptin were not found to significantly increase the risk for HF [6–9], saxagliptin has been reported to significantly increase the risk of HF [5]. On the contrary, SGLT-2i, another second-line drug for DM, has been reported to lower the risk of HF [10–12]. In addition to randomized controlled trials, several studies using real-world evidence in actual clinical environments have reported such trends [20–26].

The risk of HF among patients with DM is an important determining factor for choosing drugs in clinical practice. Reduced HF risk, as a clinical outcome of drug therapy, is associated not only with improved quality of life but also with lower medical costs. As the soaring medical cost is affecting governments’ and hospitals’ decision-making process, cost must be taken into consideration while making choices in a clinical setting. Both clinical and economic outcomes have become an important factor for choosing medications for the highly prevalent DM in today’s aging society.

Economical evaluation of DPP-4i and SGLT-2i, including dapagliflozin, as a second-line therapy for DM in the UK and US has shown that SGLT-2i is cost-effective and reduces the medical costs [27–29]. However, the results of pharmaco-economical evaluations vary according to the health insurance or healthcare service systems across countries, so it is difficult to directly cite the results reported in other countries. Therefore, country-specific assessment of the economic outcomes of the new class of DM drug, dapagliflozin, is necessary.

Consequently, the present study aimed to compare the differences in the risk of hospitalization due to HF between the dapagliflozin group, which is the first in a new class of SGLT-2i licensed in Korea, and the DPP-4i group, which are the most widely used second-line drugs for T2DM, using the latest real-world nationwide population data in Korea. Additionally, we aimed to examine the impact of clinical outcomes on medical costs in the two groups from an economic perspective.

## Methods

### Data source

A population-based cohort study was conducted using the claims data between September 01, 2014 and June 30, 2018. The data were retrieved from the Health Insurance Review & Assessment services, a government-affiliated agency, which reviews and assesses healthcare costs and service quality, as well as operates healthcare information system to support research [30]. This database contains longitudinal claims information including medical diagnoses, procedures, hospitalizations, physician visits, and prescription records of approximately 50 million Koreans. The diagnoses were coded according to the International Classification of Diseases, Tenth Revision (ICD-10).

Patients were not directly involved in the research, and only the secondary electronic database was used for the analysis. Informed consent was not required due to the retrospective nature of the study and the database maintained the anonymity of sampled individuals. This study was approved by the Cha University Institutional Review Board (Protocol ID: 1044308-201812-HR-060-01).

### Study population

Patients were eligible for inclusion in the study if they met the following criteria: had a diagnosis of T2DM (ICD-10, E11) and were aged 18–75 years between September 01, 2015 and August 31, 2016, and had ≥ 1 prescription of dapagliflozin or DPP-4i during the same period. The date of first prescription of dapagliflozin or DPP-4i was considered as the entry date in the study cohort. To include new users of dapagliflozin or DPP-4i, the patients who had been prescribed dapagliflozin or DPP-4i within 365 days prior to the cohort entry (baseline period) were excluded. Patients using other SGLT-2i and GLP-1 receptor agonists during the baseline period were also excluded. Patients with acute cardiovascular event (e.g., hospitalization with a diagnosis of HF, myocardial infarction, and ischemic stroke) within 8 weeks prior to the cohort entry were excluded. We also excluded all patients with a diagnosis of cancer, human immunodeficiency virus infection, or end stage renal disease at any time prior to the cohort entry, including those with entire period of follow-up after the cohort entry. Patients with a diagnosis of type 1 DM or gestational diabetes during the baseline period were also excluded.

### Comparison of clinical outcomes

The primary outcome was the first incidence of hospitalization for HF (hHF) (admission with ICD-10 code I50) after the index date. Each patient was followed-up from cohort entry until the first incidence of the following: hHF, treatment switch or discontinuation, i.e., a gap of > 30 days between prescription fill dates, death from any cause, or the end of the study duration (June 30, 2018).

The baseline characteristics, including sex and age, were assessed for patients in each group. To address comorbidities, patients with one of the following diagnoses within a year prior to the date of cohort entry were identified: microvascular complications of diabetes, including nephropathy, neuropathy, retinopathy; hypertension; dyslipidemia; chronic kidney disease; CVD, including myocardial infarction, other ischemic heart disease, ischemic stroke, hemorrhagic stroke, peripheral artery occlusive disease, coronary revascularization procedures (coronary artery bypass graft, percutaneous coronary intervention), HF, and atrial fibrillation; hypoglycemia; asthma; chronic obstructive pulmonary disease; connective tissue disease; pancreatitis; osteoporosis; alcohol intake; smoking habit; and obesity. For concomitant medications, patients with one of the following drug prescriptions within 180 days prior to the date of cohort entry were identified: anti-hyperglycemic agents, including metformin, sulfonylurea, thiazolidinediones, alpha-glucosidase inhibitor, meglitinide, insulin; diuretics, including loop diuretics, thiazide, aldosterone antagonist, potassium sparing diuretics; anti-hypertensive agents, including calcium channel blocker, angiotensin converting enzyme inhibitor, angiotensin II receptor blocker, beta blocker, alpha blocker; digoxin; aspirin; P2Y12 inhibitor; warfarin; non-vitamin K antagonist oral anticoagulant; and lipid-lowering agents, including statin, fibrate, ezetimibe. Further, the following healthcare utilization data were identified as covariates: visit to cardiology within 30 days prior to the date of cohort entry; hospitalization within 30 days prior to the date of cohort entry; hospitalization within 30–365 days prior to the date of cohort entry; visit to the emergency department within 365 days prior to the date of cohort entry.

Standardized differences were used to examine the baseline differences between the dapagliflozin group and DPP-4i group [31]. The rate of hHF was computed for each group. The hazard ratios (HR) and 95% confidence interval (CI) for hHF in the dapagliflozin group and DPP-4i group were computed using the Cox proportional hazards model. The incidence of hHF in each group was shown using the Kaplan–Meier curve. The baseline differences between the two groups were adjusted using propensity scores. To count for the odds for dapagliflozin to be prescribed, propensity scores were computed using the information of all covariates evaluated as the baseline characteristics—age, sex, comorbidities, concomitant medications and healthcare utilization. Propensity score-weighted analysis was performed to create a pseudo-population with equal distribution of covariates between the DPP-4i group and dapagliflozin group; these pseudo-populations of the DPP-4i and dapagliflozin groups were compared to compute the risk of hHF in each group [32–34]. Subgroup analysis was performed to analyze the risk of an event according to cardiovascular comorbidity prior to the date of cohort entry. During the sensitivity analysis, we performed intention-to-treat analysis, which assumes that the drugs prescribed at the time of cohort entry were used throughout the follow-up period, to compute the risk of hHF.

### Comparison of economic outcomes

**Fig. 1:**
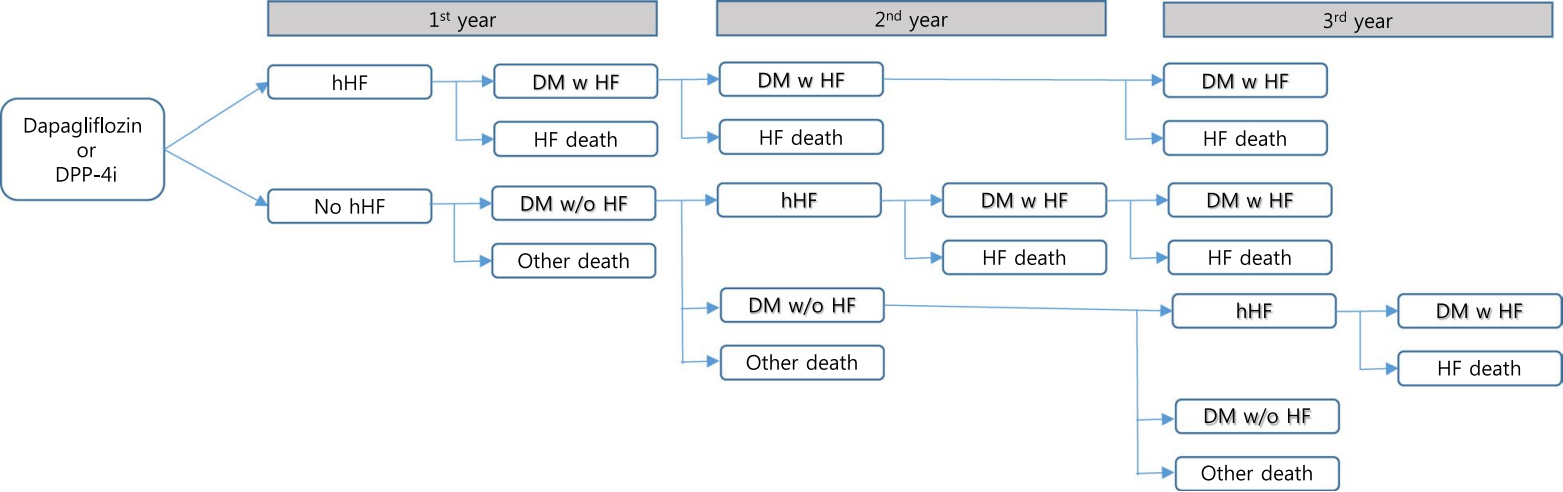
Schematic diagram of health status for cost analysis. *DPP-4i* dipeptidyl peptidase-4 inhibitors, *hHF* hospitalization for heart failure, *DM w HF* diabetes mellitus with heart failure, *DM w/o HF* diabetes mellitus without heart failure, *HF death* death after diagnosis of heart failure, *other death* death without diagnosis of heart failure

To analyze direct medical costs that reflect clinical outcomes, we used a model that incorporated changes in the patients’ health status over 3 years (Fig. 1). This model was simulated using annual medical cost corresponding to each health status and health status transition probability. We analyzed medical costs for 3 years by including 1000 participants for each cohort in the study.

In total, six health statuses were used in this model: hHF: first incidence of hHF; no hHF: no onset of hHF; DM with HF: survived from hHF and is continuing DM and HF treatment; HF death: death following hHF; DM without HF: survived without the onset of HF and is continuing DM treatment; and Other death: death without diagnosis of HF.

Health status transition probability was calculated using the HR obtained from clinical outcome analysis. The incidence rate of hHF for DPP-4i group was used to convert to 1-year probability, and that for the dapagliflozin group was calculated by multiplying HR to the incidence rate of the DPP-4i group. For estimating the probability of mortality based on the diagnosis of hHF, the mortality rate of DPP-4i group was calculated by following-up the participants from cohort entry to death or study termination (June 2018), and the HR for death for the dapagliflozin group was computed against the death rate of DPP-4i group using propensity score weighting.

Based on the follow-up of the entire study population from cohort entry until death or study termination (June 2018), the median per patient medical cost/month was identified. Medical costs were computed according to the occurrence of an event during follow-up and death/survival. The annual medical cost of survivors continuing the treatment was calculated by multiplying the monthly treatment cost by 12, and that of dead patients was calculated by multiplying the monthly treatment cost by 6, under the assumption that they survived for 6 months in a year. Further, as we used insurance claims data for this analysis, we applied the non-coverage rate by disease to estimate patients’ out-of-pocket costs. The non-coverage rate for patients with heart disease as of 2018 (6.3%) was applied for the hHF patients, and the non-coverage rate for the entire health insurance recipient population (16.6%) was applied for the non-hHF patients [35]. Cost is presented as US dollars, which was calculated on the basis of annual average currency exchange rate for 2018 (1165 KRW = 1$).

The incidence of hHF among patients undergoing treatment was utilized for basic analysis, while the incidence of hHF and HR of intend-to-treat patients was used for the sensitivity analysis. Analyses were performed using the SAS 9.4 version (SAS Institute Inc, Cary, NC, USA), and survival curves were drawn using R software 3.1.2 version (R Foundation for Statistical Computing, Vienna, Austria).

## Results

### Study population and patient characteristics

**Fig. 2:**
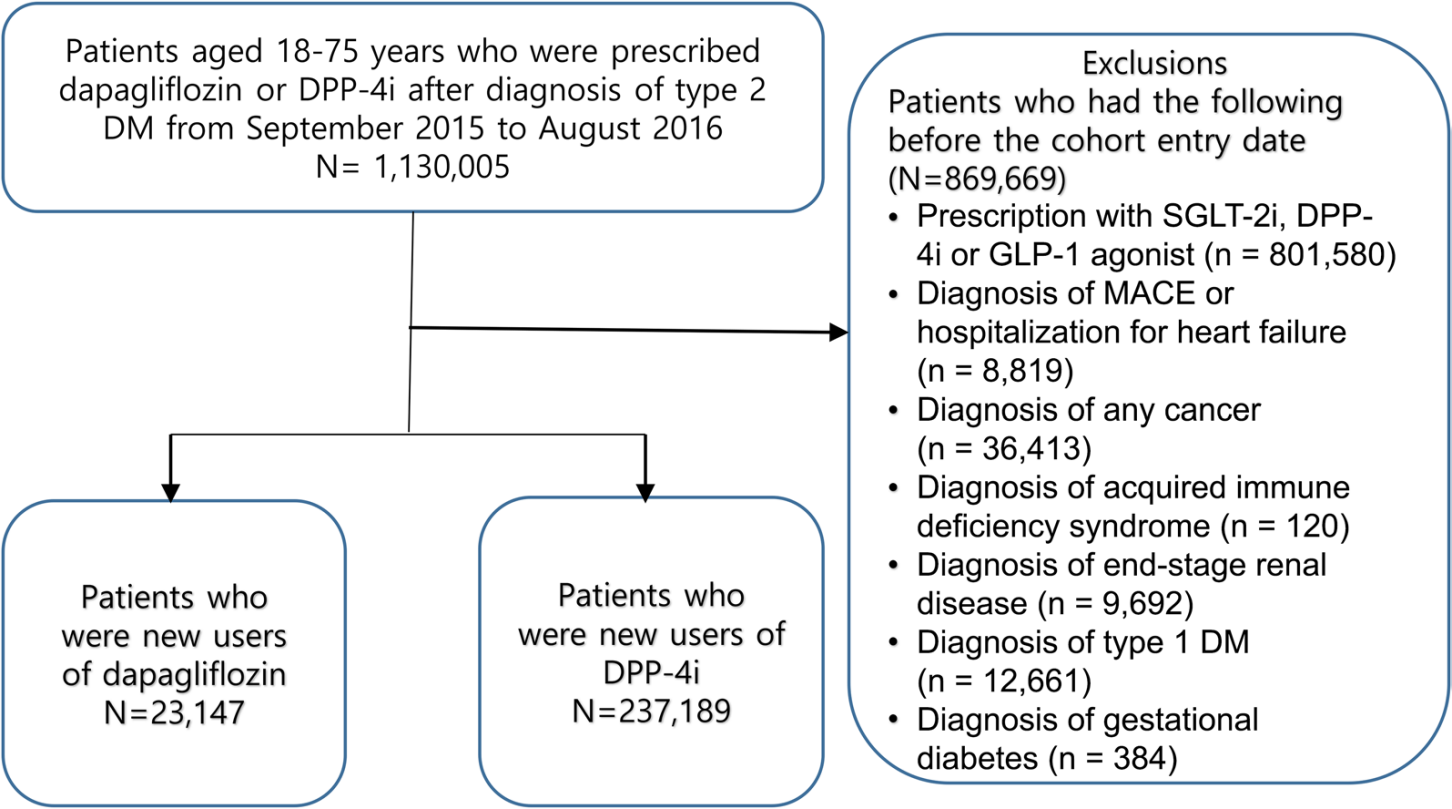
Flow chart for sample selection. *DPP-4i* dipeptidyl peptidase-4 inhibitors, *DM* diabetes mellitus, *SGLT-2i* sodium glucose co-transporter 2 inhibitor, *GLP-1* glucagon‐like peptide 1, *MACE* major adverse cardiovascular event

In total, 1,130,005 patients were prescribed DPP-4i or dapagliflozin after the diagnosis of T2DM during the recruitment period from September 2015 to August 2016. After excluding 869,669 patients per the exclusion criteria, 23,147 patients were new dapagliflozin users, and 237,187 patients were new DPP-4i users (Fig. 2).

Prior to the propensity score weighting, dapagliflozin group had younger participants and a lower percentage of men as compared to the DPP-4i group. The number of patients with a history of admission within 30 days prior to the index date was greater in the DPP-4i group, while the percentage of patients who recently visited a cardiologist was greater in the dapagliflozin group. Dyslipidemia was the only comorbidity that showed a significant difference at baseline between the two groups, with an SD of ≥ 10%, and the prevalence of dyslipidemia was higher in the dapagliflozin group than in the DPP-4i group. The percentage of patients using a statin was also higher in the dapagliflozin group as compared to the DPP-4i group. Though the number of patients taking anti-hyperglycemic medications was higher in the DPP-4i group, the number of patients using insulin was higher in the dapagliflozin group. However, all variables were well balanced between the two groups after propensity score weighting (Table 1).

**Table 1:** Baseline characteristics of the study population before and after propensity score weighting

	Entire population			Propensity score weighted population		
	Dapagliflozin (N = 23,147)	DPP-4i (N = 237,189)	Standardized difference	Dapagliflozin (N = 23,147)	DPP-4i (N = 237,189)	Standardized difference
Age (years), mean (SD)	52.6 (11.50)	56.9 (10.88)	0.38	52.6 (11.50)	52.7 (3.59)	0.00
Men	12,557 (0.54)	141,542 (0.60)	0.11	12,557 (0.54)	12,584 (0.54)	0.00
Microvascular complication of diabetes						
Nephropathy	1951 (0.08)	15,372 (0.06)	0.07	1951 (0.08)	1970 (0.09)	0.00
Neuropathy	2836 (0.12)	26,453 (0.11)	0.03	2836 (0.12)	2840 (0.12)	0.00
Retinopathy	3641 (0.16)	30,890 (0.13)	0.08	3641 (0.16)	3623 (0.16)	0.00
Cardiovascular disease						
Hemorrhagic stroke	86 (0.00)	1495 (0.01)	0.04	86 (0.00)	85 (0.00)	0.00
Other ischemic heart disease	3498 (0.15)	28,245 (0.12)	0.09	3498 (0.15)	3521 (0.15)	0.00
Ischemic stroke	752 (0.03)	10,022 (0.04)	0.05	752 (0.03)	755 (0.03)	0.00
Myocardial infarction	291 (0.01)	2287 (0.01)	0.03	291 (0.01)	294 (0.01)	0.00
Heart failure	817 (0.04)	6895 (0.03)	0.04	817 (0.04)	833 (0.04)	0.00
Peripheral artery occlusive disease	158 (0.01)	1875 (0.01)	0.01	158 (0.01)	160 (0.01)	0.00
Coronary artery bypass graft	6 (0.00)	107 (0.00)	0.01	6 (0.00)	6 (0.00)	0.00
Percutaneous coronary intervention	407 (0.02)	3552 (0.01)	0.02	407 (0.02)	417 (0.02)	0.00
Comorbidities^a^						
Obesity	103 (0.00)	447 (0.00)	0.05	103 (0.00)	100 (0.00)	0.00
Osteoporosis	1602 (0.07)	21,719 (0.09)	0.08	1602 (0.07)	1605 (0.07)	0.00
Atrial fibrillation	370 (0.02)	3829 (0.02)	0.00	370 (0.02)	376 (0.02)	0.00
Hypertension	13,992 (0.60)	135,909 (0.57)	0.06	13,992 (0.60)	14,001 (0.61)	0.00
Alcohol intake	1014 (0.04)	13,260 (0.06)	0.06	1014 (0.04)	1016 (0.04)	0.00
Smoking habit	38 (0.00)	223 (0.00)	0.02	38 (0.00)	38 (0.00)	0.00
Asthma	2914 (0.13)	28,920 (0.12)	0.01	2914 (0.13)	2911 (0.13)	0.00
Chronic kidney disease	167 (0.01)	3583 (0.02)	0.08	167 (0.01)	171 (0.01)	0.00
COPD	1092 (0.05)	12,879 (0.05)	0.03	1092 (0.05)	1096 (0.05)	0.00
Connective tissue disease	841 (0.04)	9117 (0.04)	0.01	841 (0.04)	844 (0.04)	0.00
Pancreatitis	374 (0.02)	3999 (0.02)	0.01	374 (0.02)	367 (0.02)	0.00
Hypoglycemia	535 (0.02)	5450 (0.02)	0.00	535 (0.02)	534 (0.02)	0.00
Dyslipidemia	19,361 (0.84)	183,745 (0.77)	0.16	19,361 (0.84)	19,342 (0.84)	0.00
Medication use						
Anti-diabetic agent						
Number of drugs	2.0 (0.64)	2.1 (0.64)	0.24	2.0 (0.64)	2.0 (0.20)	0.00
Metformin	19,334 (0.84)	211,407 (0.89)	0.16	19,334 (0.84)	19,313 (0.83)	0.00
Sulfonylurea	8859 (0.38)	102,332 (0.43)	0.10	8859 (0.38)	8907 (0.39)	0.00
Thiazolidinediones	2508 (0.11)	21,575 (0.09)	0.06	2508 (0.11)	2512 (0.11)	0.00
Meglitinide	198 (0.01)	1724 (0.01)	0.01	198 (0.01)	202 (0.01)	0.00
α-glucosidase inhibitor	967 (0.04)	10,930 (0.05)	0.02	967 (0.04)	984 (0.04)	0.00
Insulin	4456 (0.19)	32,334 (0.14)	0.15	4456 (0.19)	4438 (0.19)	0.00
Anti-hypertensive drugs						
Calcium channel blocker	6356 (0.27)	64,718 (0.27)	0.00	6356 (0.27)	6337 (0.27)	0.00
ACEI	578 (0.02)	4238 (0.02)	0.05	578 (0.02)	590 (0.03)	0.00
β-blocker	2101 (0.09)	18,425 (0.08)	0.05	2101 (0.09)	2111 (0.09)	0.00
ARB	10,482 (0.45)	100,108 (0.42)	0.06	10,482 (0.45)	10,466 (0.45)	0.00
α-blocker	154 (0.01)	1546 (0.01)	0.00	154 (0.01)	153 (0.01)	0.00
Diuretics						
Thiazide	3178 (0.14)	31,487 (0.13)	0.01	3178 (0.14)	3166 (0.14)	0.00
Aldosterone antagonist	445 (0.02)	4181 (0.02)	0.01	445 (0.02)	452 (0.02)	0.00
Loop diuretics	765 (0.03)	9539 (0.04)	0.04	765 (0.03)	777 (0.03)	0.00
Potassium sparing diuretics	13 (0.00)	155 (0.00)	0.00	13 (0.00)	13 (0.00)	0.00
Warfarin	128 (0.01)	1461 (0.01)	0.01	128 (0.01)	133 (0.01)	0.00
NOAC	98 (0.00)	1384 (0.01)	0.02	98 (0.00)	101 (0.00)	0.00
Aspirin	4872 (0.21)	51,286 (0.22)	0.01	4872 (0.21)	4909 (0.21)	0.00
P2Y12 inhibitor	2643 (0.11)	23,930 (0.10)	0.04	2643 (0.11)	2667 (0.12)	0.00
Digoxin	145 (0.01)	1845 (0.01)	0.02	145 (0.01)	147 (0.01)	0.00
Lipid-lowering agents						
Statin	12,354 (0.53)	113,483 (0.48)	0.11	12,354 (0.53)	12,358 (0.53)	0.00
Ezetimibe	1556 (0.07)	11,881 (0.05)	0.07	1556 (0.07)	1559 (0.07)	0.00
Fibrate	1447 (0.06)	11,964 (0.05)	0.05	1447 (0.06)	1454 (0.06)	0.00
Healthcare utilization						
Cardiologist visit	2116 (0.09)	13,155 (0.06)	0.14	2116 (0.09)	2172 (0.09)	0.01
Emergency department visit	1758 (0.08)	18,209 (0.08)	0.00	1758 (0.08)	1761 (0.08)	0.00
Hospitalization (within 30 days)^b^	1506 (0.07)	29,675 (0.13)	0.21	1506 (0.07)	1509 (0.07)	0.00
Hospitalization (during 30–365 days)^c^	3524 (0.15)	37,400 (0.16)	0.02	3524 (0.15)	3548 (0.15)	0.00

Data are presented as frequency (percentage), or mean (standard deviation)

*DPP-4i* Dipeptidyl-peptidase 4 inhibitor, *SD* standard deviation, *COPD* chronic obstructive pulmonary disease, *ACEI* angiotensin-converting-enzyme inhibitor, *ARB* angiotensin-2 receptor antagonist, *NOAC* Novel oral anticoagulant

^a^Confirmed by diagnosis code (International Classification of Diseases, 10th revision)

^b^Hospitalization within 30 days prior to index date

^c^Hospitalization during 30–365 days prior to index date

### Comparison of clinical outcomes

**Fig. 3:**
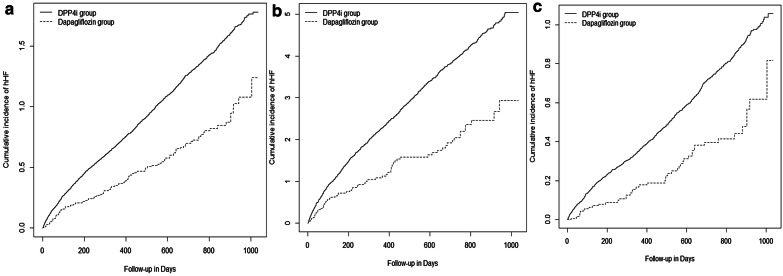
Kaplan–Meier plots of hospitalization for heart failure in all patients (**a**), and baseline cardiovascular stratifications with underlying cardiovascular disease (**b**), and without underlying cardiovascular disease (**c**). *DPP-4i* dipeptidyl peptidase-4 inhibitor, *hHF* hospitalization for heart failure

The total number of hHF events during an average follow-up period of 16.3 months (dapagliflozin 14.7 months, DPP-4i 16.5 months) was 4537; and the incidence rate in the dapagliflozin and DPP-4i groups was 3.86 and 6.79 per 1000 person-year, respectively. In the entire study population, the adjusted HR (aHR) for hHF in the dapagliflozin group as compared to the DPP-4i group was 0.58 (95% CI 0.46–0.74), and was 0.55 (95% CI 0.41–0.74) among patients with underlying CVD and 0.66 (95% CI 0.46–0.95) among patients without underlying CVD (Table 2 and Fig. 3). In the intend-to-treat analysis, the dapagliflozin group also had a significantly lower risk for hHF (aHR: 0.70, 95% CI 0.60–0.82) (Table 2) than the DPP-4i group.

**Table 2:** The risk of hospitalization for heart failure of dapagliflozin group compared with DPP-4i group

	Dapagliflozin group			DPP-4i group			cHR	95% CI	aHR	95% CI
	No. of events	Person-year (PY)	Incidence (/1000PY)	No. of events	Person-year (PY)	Incidence (/1000PY)				
On treatment analysis										
Total	110	28,478	3.86	2210	325,344	6.79	0.56	0.46–0.68	0.58	0.46–0.74
CVD	64	5605	11.42	1196	57,819	20.69	0.54	0.42–0.69	0.55	0.41–0.74
Non-CVD	46	22,873	2.01	1014	267,525	3.79	0.53	0.39-.071	0.66	0.46–0.95
Intention-to-treat analysis										
Total	267	54,145	4.93	4270	546,982	7.81	0.63	0.56–0.71	0.70	0.60–0.82
CVD	148	10,657	13.89	2084	93,600	22.26	0.62	0.53–0.74	0.66	0.53–0.81
Non-CVD	119	43,488	2.74	2186	453,382	4.82	0.57	0.47–0.68	0.74	0.58–0.94

*DPP-4i* Dipeptidyl-peptidase 4 inhibitor, *PY* person-year, *cHR* crude hazard ratio, *aHR* adjusted hazard ratio, *CI* confidence interval, *CVD* Patients with underlying cardiovascular disease, *Non-CVD* patients without underlying cardiovascular disease

### Comparison of economical outcomes

The annual average treatment cost for patients who survived after hHF and continued treatment was $4964 per patient, which was approximately six times higher than that for patients without hHF. The annual medical cost for patients who died after hHF was $6718 per patient, which was approximately 2.3 times higher than that for patients who died without hHF (Table 3). Table 4 shows the parameters used in the cost analysis model that utilized HR for hHF and death probabilities according to hHF. Direct medical costs, calculated using the model for 1000 patients per drug group, were $2,542,221 for the dapagliflozin group and $2,600,008 for the DPP-4i group over 3 years, thereby indicating that the cost was $57,787 lower in the dapagliflozin group. The difference increased to $177,580 among patients with underlying CVD, which was approximately seven times higher than that among the patients without underlying CVD (Table 5). In the sensitivity analysis using the HR of intend-to-treat patients, the direct medical cost was $48,097 lower in the dapagliflozin group over 3 years, and the reduction of direct medical cost was approximately 5.7 times greater among patients with underlying CVD as compared to those without underlying CVD (Table 5).

**Table 3:** Medical cost according to health status (USD/year/person)

Patient groups	Health status	Annual direct medical cost (USD)
All patients	DM with HF	4964.0
	HF death	6718.1
	DM without HF	813.6
	Other death^a^	2919.5
Patients with underlying CVD	DM with HF	5321.5
	HF death	6615.5
	DM without HF	1286.5
	Other death^a^	2783.9
Patients without underlying CVD	DM with HF	4701.1
	HF death	6864.5
	DM without HF	743.2
	Other death^a^	2964.6

Data source: Claim data of the Health Insurance Review & Assessment services

*DM* Diabetes mellitus, *HF* heart failure, *CVD* cardiovascular disease

^a^ Death without diagnosis of heart failure

**Table 4:** Model inputs: probabilities of hospitalization for heart failure and death for dapagliflozin/DPP-4i treated patients

Patient groups	Base analysis		Sensitivity analysis	
	Dapagliflozin	DPP-4i	Dapagliflozin	DPP-4i
All patients				
hHF incidence	0.0054	0.0078	0.0040	0.0068
HF death	0.0198	0.0253	0.0198	0.0253
Other death	0.0010	0.0013	0.0010	0.0013
Patients with underlying CVD				
hHF incidence	0.0145	0.0220	0.0113	0.0205
HF death	0.0105	0.0243	0.0105	0.0243
Other death	0.0016	0.0024	0.0016	0.0024
Patients without underlying CVD				
hHF incidence	0.0036	0.0048	0.0025	0.0038
HF death	0.0355	0.0264	0.0355	0.0264
Other death	0.0009	0.0010	0.0009	0.0010

Data source: Claim data of the Health Insurance Review & Assessment services

*DPP-4i* Dipeptidyl-peptidase 4 inhibitor, *hHF* hospitalization for heart failure, *HF* heart failure, *CVD* cardiovascular disease

**Table 5:** Direct medical costs (USD) for 3 years among dapagliflozin treated patients and DPP-4i treated patients

Patient groups	Treatment	Treatment year			Total
		1st year	2nd year	3rd year	
Base analysis					
All patients	Dapagliflozin	832,312	847,560	862,349	2,542,221
	DPP-4i	844,608	870,883	884,517	2,600,008
	Difference	12,296	23,323	22,169	57,787
Patients with underlying CVD	Dapagliflozin	1,334,443	1,376,727	1,417,845	4,129,014
	DPP-4i	1,373,319	1,448,896	1,484,380	4,306,594
	Difference	38,876	72,169	66,535	177,580
Patients without underlying CVD	Dapagliflozin	755,451	764,393	772,895	2,292,739
	DPP-4i	760,709	774,577	782,840	2,318,126
	Difference	5258	10,184	9945	25,387
Sensitivity analysis					
All patients	Dapagliflozin	838,482	859,691	880,237	2,578,409
	DPP-4i	848,827	879,127	898,553	2,626,506
	Difference	10,345	19,436	18,317	48,097
Patients with underlying CVD	Dapagliflozin	1,347,676	1,402,655	1,455,945	4,206,275
	DPP-4i	1,379,600	1,460,981	1,508,597	4,349,179
	Difference	31,925	58,327	52,653	142,904
Patients without underlying CVD	Dapagliflozin	759,669	772,623	784,938	2,317,229
	DPP-4i	764,829	782,650	794,789	2,342,268
	Difference	5160	10,027	9851	25,039

*DPP-4i* Dipeptidyl-peptidase 4 inhibitor, *CVD* cardiovascular disease

## Discussion

The present study showed that dapagliflozin lowered the risk for hHF and subsequently lowered direct medical costs as compared to DPP-4i in patients with T2DM.

Two large cardiovascular outcomes trials (CVOTs) of SGLT-2i—Empagliflozin Cardiovascular Outcome Event Trial in Type 2 Diabetes Mellitus Patients (EMPA-REG OUTCOME) and Canagliflozin Cardiovascular Assessment Study (CANVAS)—demonstrated consistent reduction in the incidence of hHF among patients with T2DM, although hHF was not a primary endpoint in these trials [10, 11]. In the Dapagliflozin Effect on Cardiovascular Events–Thrombolysis in Myocardial Infarction 58 (DECLARE-TIMI 58) study, treatment with dapagliflozin resulted in a lower rate of cardiovascular death or hHF (one of the primary outcomes) in patients with T2DM [12]. Moreover, a recent meta-analysis of these 3 CVOTs showed that SGLT-2i reduced the risk of hHF in patients with T2DM regardless of the presence of CVD or history of HF [36]. Previous CVOTs of SGLT-2i included T2DM patients with established CVD or at the high risk for CVD. However, the present study covered a wider range of nationwide population cohort including patients with and without CVD. Our results showed that dapagliflozin reduced the risk for hHF in patients with and without CVD, and the HF protective effect of dapagliflozin was more evident among patients with underlying CVD.

In the present study, we compared dapagliflozin, a first-in-class SGLT-2i, with DPP-4i, one of the most widely used second-line oral anti-hyperglycemic agents in a real-world observational cohort. The findings of our study showed that the use of dapagliflozin reduced hHF as compared to DPP-4i. Our results are consistent with the results of previous observational studies, wherein, SGLT-2i was compared with other non-SGLT-2i oral anti-hyperglycemic agents [20–23], sulfonylureas and DPP-4i [24], or DPP-4i in a similar cohort [25]. A large Scandinavian cohort study also reported that SGLT-2i use compared with DPP-4i use was associated with a reduced risk of HF, with similar HR to that in our study both in on-treatment analysis and intention-to-treat analysis. Moreover, the magnitude of the protective association between SGLT-2i and HF was larger in on-treatment analysis, suggesting that the HF protective effect of SGLT-2i could be stronger during the time that patients stay on the drug [26]. A network meta-analysis to compare the effect on CV outcomes among SGLT-2i, GLP-1 receptor agonist, and DPP-4i also reported that SGLT-2i show clear superiority in reducing hHF among the three new drug classes [37].

Our data provide information on the risk of hHF of dapagliflozin, not of all SGLT2i. The effects on hHF may differ between individual SGLT-2i, because not all SGLT-2i share the same pharmacokinetic properties [38]. A retrospective cohort study reported that dapagliflozin users had a significantly lower risk of HF as compared to empagliflozin users, and further studies are required to confirm the findings [39].

A recent placebo-controlled trial (DAPA-HF trial) showed that the risk of worsening HF or death from cardiovascular causes was lower among the HF patients with reduced ejection fraction who received dapagliflozin than those who received placebo, regardless of the presence or absence of diabetes [40]. Moreover, dapagliflozin reduced hHF both in patients with and without HFrEF in the stratified analysis by baseline ejection fraction of DECLARE-TIMI 58 patients. [41]. Thus, the growing evidence is suggestive of HF protective effects of SGLT-2i. Several underlying mechanisms have been proposed to explain the HF protective effect of SGLT-2i, such as improvement in ventricular loading conditions through a reduction in preload and afterload, improvement in cardiac metabolism and bioenergetics, myocardial Na^+^/H^+^ exchange inhibition, reduction of necrosis and cardiac fibrosis and an alteration in adipokine production [42].

The recent consensus report by the American Diabetes Association (ADA) and the European Association for the Study of Diabetes (EASD) addresses the approaches to management of glycemia in patients with T2DM, with the goal of reducing complications and maintaining quality of life in the context of comprehensive cardiovascular risk management changed from the prior consensus statements, wherein, efficacy in reducing hyperglycemia, along with tolerability and safety were primary factors in glucose-lowering medication selection [16]. The recently updated consensus report by the ADA and the EASD suggested that SGLT-2i are recommended in patients with T2DM and HF, particularly those with HF with HFrEF, to reduce hHF, major adverse cardiovascular event, and CVD death [43]. Our study showed the superiority of dapagliflozin to DPP-4i, one of the most widely used oral hypoglycemic agents, in terms of HF protection in a real-world clinical setting and broad T2DM population, and in accordance with the current guideline, prior CVOTs, meta-analyses, and observation studies.

Our economical evaluation, based on a simulation on 1000 patients for each group, showed that the dapagliflozin group saved about $12,000 of medical costs in the first year, with a two-fold greater cost reduction in the second year as compared to the DPP-4i group. With the use of dapagliflozin, approximately $58,000 of medical cost was saved over 3 years, with a threefold greater cost reduction among patients with underlying CVD. Similar results were found in the sensitivity analysis on intend-to-treat patients, thereby confirming that dapagliflozin is highly effective in lowering hHF risk and cutting medical costs in patients with underlying CVD.

An analysis for cost-effectiveness of dapagliflozin, conducted in the UK, showed that dapagliflozin had an incremental cost-effectiveness ratio (ICER) of ₤6761 per quality adjusted life years (QALY) gained as compared to DPP-4i; based on which, dapagliflozin was found to be a cost-effective agent as an addition to the regimen for patients with DM whose blood glucose was not appropriately controlled with only metformin as compared to DPP-4i, which was the most widely used agent for the purpose [27]. The changes in HbA1c level and body weight for 1 year were used as the parameters for comparing clinical outcomes between the dapagliflozin and DPP-4i groups, and the authors claimed that the superior weight-loss effects of dapagliflozin were the underlying reasons for increased QALY. In a simulation of 30,000 patients for 40 years using a model, including the probability of DM complications in relation to weight loss and related QALY and cost, dapagliflozin was associated with ₤216 more cost and 0.032 increase of QALYs compared to DPP-4i. These findings were obtained on the basis of long-term simulation with weight loss as the major outcome amid a lack of long-term clinical outcome data and data on major DM complications associated with dapagliflozin and DPP-4i, which may lead to a difference from the results that reflect the real-world reports of CVD and renal complications. In contrast, we analyzed a simplified model for a relatively short time period (3 years), reflecting the clinical outcomes and cost values measured through real-world data analysis to minimize the influence of the limitations of economic analysis, such as excessive extrapolation and assumption.

In another economic evaluation conducted in Australia, decision analysis was applied to assess the cost-effectiveness of first-line combination dapagliflozin and metformin (first-line use) versus first-line metformin monotherapy followed by gradual addition of dapagliflozin over time (delayed use) [28]. Clinical outcomes were derived from a published observational study, CVD-REAL Nordic, and a Markov model was used to simulate the progress for 20 years. The Australia study showed that first-line use had an ICER of AUD $12,477 per QALY gained as compared to delayed use, even though first-line use of the combination is not recommended in the current guidelines [28]. Both UK and Australian studies have shown that dapagliflozin is not only effective compared to DPP-4i or delayed use, but also increases costs. This study did not analyze the ratio of the cost to the effectiveness, but showed the reduced the medical cost due to the improved clinical outcomes. Therefore, care should be taken to quantitatively compare and interpret the results of this study with other CE analysis.

Further, Garry et al. analyzed the medical costs between patients who used SGLT-2i and patients who used DPP-4i as second-line therapy using insurance claims data in the US and reported that the annual total cost of care was $3419 (95% CI − $11,264 to − $4426) lower among patients in the CVD high risk group who used SGLT-2i [29]. The patients were classified according to the first drug used in the second-line therapy, and a pairwise comparison was performed between the SGLT-2i and DPP-4i groups using adjusted deciles of propensity score and Cox proportional hazards regression model, with treatment costs compared without any simulation. Studies that compare costs related to the use of specific drugs produce markedly different outcomes depending on the structure of the model, variables used, and the method of computing parameters. In our study, we focused only on hHF, the primary clinical outcome related to dapagliflozin using real-world data and the total cost was estimated using a simple model simulation by analyzing the cost incurred according to the incidence of hHF as opposed to the actual cost per drug group. Currently, five SGLT-2is—dapagliflozin, ipragliflozin, empagliflozin, canagliflozin, and ertugliflozin—are available in Korea; however, dapagliflozin was the only drug included in the national health insurance formulary during the patient enrollment period of this study, from September 01, 2015 to August 31, 2016. In the early days following the introduction of a novel class of drugs in clinical practice, treatment decisions are made on the basis of clinical trials of the drugs, so patients make prudent selections. Inevitably, there are differences between patients who use DPP-4i, which has significantly accumulated usage data, and those who choose dapagliflozin, which is a novel drug. Even if these differences are adjusted for statistically, the influence of unmeasured confounders cannot be completely eliminated. Moreover, as using insurance claims data is bound by limitations such as lack of detailed clinical documentation, misdiagnosis, and miscoding; it may be challenging to identify unmeasured confounders. Therefore, we used direct measurements of cost according to the incidence of hHF as the model parameter to minimize such influence, and for this reason, our model simulation computed cost incurred according to the risk for hHF by drug group.

A CVD event is a major cause of elevated medical cost for patients with DM. Garry et al. reported that among patients with high CVD risk, the total cost of care was higher for DPP-4i users than for dapagliflozin users [29], and our study also found that the difference in cost was higher among patients with underlying CVD. Thus, dapagliflozin may have maximal economic value when administered in patients with a high risk of a CVD event.

It is common for a typical economic evaluation to help decision making by presenting an increased amount of cost as a ratio as well as an increased effect (ICER) between alternatives. However, in this study, the analysis based on real-world data showed that dapagliflozin has an effect of reducing risk of hHF compared to DPP-4i, and even economical results of reducing medical costs, even though only by 2%. Ultimately, it supports that dapagliflozin is a dominant treatment strategy compared to DPP-4i. In countries with different health care systems, there may be quantitative differences in the economic performance of the same comparative alternative. This study is meaningful in that it estimated the amount of direct medical cost reduction due to a reduction in hHF risk in certain health care systems in Korea.

Our study had a few limitations. First, because it was a retrospective observational study, residual confounding factors could not be completely excluded, although we used propensity score weighting to adjust effects of confounders. In addition, our national claims data do not contain information concerning demographics such as diabetes duration, body mass index, or laboratory test results. Second, we relied on diagnostic codes for outcome ascertainment. However, a validation study reported that the overall positive predictive value of the ICD-10 codes was approximately 70%, compared with medical records reviews [44]. Moreover, mortality data were analyzed on the basis of diagnosis codes for the meddling, so it may differ from the actual mortality data. Third, this study used only hHF as the major clinical outcome and only compared direct medical costs incurred over 3 years. However, there are several other complications that contribute to the treatment cost of patients with DM, such as myocardial infarction, stroke, kidney complication, and premature death; and the duration of DM spans several decades. A long-term analysis including these major clinical outcomes need to be performed to more comprehensively compute the economic outcomes of a specific drug in order for the findings to serve as useful evidence for determining treatment strategies in clinical practice and devise health insurance financial policies.

## Conclusions

In conclusion, this study showed that dapagliflozin lowers the risk for hHF and subsequently, reduces direct medical costs as compared to DPP-4i. The protective effect against hHF was more evident among patients with underlying CVD.

## Data Availability

The datasets analyzed in this study are available from the database of Healthcare Bigdata Hub in Korean Health Insurance Review and Assessment Service (https://opendata.hira.or.kr/home.do).

## Abbreviations

ACEI: Angiotensin converting enzyme inhibitor
aHR: Adjusted hazard ratio
ARB: Angiotensin-2 receptor antagonist
cHR: Crude hazard ratio
CI: Confidence interval
COPD: Chronic obstructive pulmonary disease
CVD: Cardiovascular disease
CVOT: Cardiovascular outcomes trials
DM: Diabetes mellitus
DPP-4i: Dipeptidyl peptidase-4 inhibitors
GLP-1: Glucagon‐like peptide 1
HF: Heart failure
hHF: Hospitalization for heart failure
HFrEF: Heart failure with reduced ejection fraction
HR: Hazard ratio
NOAC: Novel oral anticoagulant
PY: Person-year
SGLT-2i: Sodium glucose co-transporter 2 inhibitors
T2DM: Type 2 diabetes mellitus
